# The Cause and Prevention of Decay in Teeth

**Published:** 1901-12

**Authors:** 


**The Cause and Prevention of Decay in Teeth.** By J. Sim Wallace, M.D. Pp. 101. London: J. and A. Churchill. 1900.

We are much indebted to Dr. Sim Wallace for giving us a book from his able contributions which have appeared from time to time in the *Journal of the British Dental Association* on "The Etiology of Dental Caries."

The headings of the chapters are sufficient to show how carefully and exhaustively he has gone into the subject, and it is not too much to say that it is a work that ought to be in the hands of every student and practitioner of dental surgery.

The author not only sets forth that dental caries is due to chemical action, but clearly explains that he is entirely against the vital action theory; and he lays great stress on diet. He says that pap and soft food collect around and between the teeth, and especially in places where there is an irregularity of the teeth arising from the overcrowding of the jaws, and that if wholesome natural food, such as whole-meal bread, had been the customary article of diet, the fibrous part of the wheat would have acted as Nature's tooth brush.

To sum up the author's arguments, we take it that decay of teeth is entirely due to the unnatural way we are fed and brought up from birth—that is, in infancy, childhood, and manhood.

We certainly agree with him that the expecting mother should live an active and healthy life, so as to be able to give her offspring natural nourishment; and that the act of suckling develops the child's tongue, and if it becomes fittingly enlarged it presses out the arches of the jaws and makes them large enough for the growth of the teeth without crowding. But if the child is fed from a bottle, the hole in the mouthpiece of the tube is generally too large; and as no effort is necessary in sucking, the tongue loses the enlargement, and in consequence the jaws are not properly developed for the accommodation of the teeth.

The diet table in the last chapter but one is well worthy of notice, as it is the result of painstaking investigations and experiments extending over many years by an able practitioner who shows a thorough knowledge of whatever relates to dental surgery. Lastly, we heartily commend this book to the attention of our professional brethren as well worthy of a careful study, and as containing much practical and valuable information.

